# Intramuscular Tendon Injuries of the Hamstring Muscles: A More Severe Variant? A Narrative Review

**DOI:** 10.1186/s40798-023-00621-4

**Published:** 2023-08-14

**Authors:** Fearghal Kerin, Stuart O’Flanagan, Joe Coyle, Garreth Farrell, Darragh Curley, Ulrik McCarthy Persson, Giuseppe De Vito, Eamonn Delahunt

**Affiliations:** 1 School of Public Health, Physiotherapy and Sports Science, University College Dublin, Dublin, Ireland; 2 Leinster Rugby, Dublin, Ireland; 3 Radiology Department, Sports Surgery Clinic, Dublin, Ireland; 4 University of Padova, Padua, Italy; 5 Institute for Sport and Health, University College Dublin, Dublin, Ireland

**Keywords:** Athletic injuries (MeSH), Soft tissue injuries (MeSH), Hamstring muscles (MeSH), Rehabilitation (MeSH)

## Abstract

Hamstring strain injuries (HSI) are one of the most common sport-related injuries. They have a high injury burden and a high recurrence rate. The development of novel muscle injury grading systems has provided new insights into the possible impact of injury location on the time to return to play (TTRTP) and re-injury following HSI. In particular, injuries to the intramuscular tendon (IMT) may be present in up to 41% of all HSI and have been described as a ‘serious thigh muscle strain’. Re-injury rates as high as 60% have been described in elite track and field athletes, as well as prolonged TTRTP. A systematic search was carried out using appropriate keywords to identify articles reporting on HSI involving the IMT in athletes. The primary aim was to determine whether IMT injuries warrant being classified as a distinct clinical entity with different expected outcomes to other hamstring muscle injuries. This narrative review summarises the existing evidence on: (1) the anatomy of the IMT and its response to injury; (2) the role of MRI and novel grading scales in IMT injury management (3) clinical assessment of IMT injuries, (4) TTRTP and re-injury rates across sports following IMT, (5) conservative rehabilitation and the role of specific ‘IMT-oriented’ strategies, and (6) indications for and approaches to surgery. The review found that important clinical outcomes such as re-injury rates and TTRTP vary across populations, cohorts and sports which suggest that outcomes are specific to the sporting context. Bespoke rehabilitation, tailored to IMT injury, has been shown to significantly reduce re-injuries in elite track and field athletes, without compromising TTRTP. Continued prospective studies across other sports and cohorts, are warranted to further establish relevant clinical findings, indications for surgical intervention and outcomes across other sporting cohorts.

**Supplementary Information:**

The online version contains supplementary material available at 10.1186/s40798-023-00621-4.

## Key Points

- Injuries to the intramuscular tendon (IMT) of the hamstrings have been proposed to associate with higher re-injury rates and longer time to return to play (TTRTP) than other hamstring strain injuries that do not involve the IMT.
- While some studies have reported longer TTRTP and higher re-injury rates following IMT disruption, other double-blinded studies have reported small or no differences in these respective outcome metrics. However, substantial homogeneity of study population cohorts, study design, sports, and approaches to rehabilitation make cross-comparison of these outcome metrics difficult.
- A bespoke “intramuscular tendon-oriented” approach to rehabilitation has been advocated in certain cohorts and sports. Such an approach has been shown to reduce re-injury rates.

**Supplementary Information:**

The online version contains supplementary material available at 10.1186/s40798-023-00621-4.

## Background

Hamstring strain injuries (HSIs) are the most commonly sustained muscle injuries in sports that involve dynamic tasks, like accelerating, over-stretching, decelerating, sprinting and kicking [1–7]. Despite recent advances in injury risk mitigation initiatives and therapeutic rehabilitation strategies [8–12], the rates of hamstring muscle injuries have increased season-on-season in the highest-level of professional European football [13]. The high prevalence of hamstring muscle injuries is compounded by high injury recurrence rates (16–34%). [3, 5, 14–18].

A history of the previous hamstring muscle injury is a well-established risk factor for future hamstring muscle injuries [19]. In professional sport, the financial cost of any injury is high [20], while player unavailability due to injury has a negative impact on a team’s performance [21, 22]. Failure to ensure an athlete’s sustained return to sport following hamstring muscle injury can undermine the athlete’s and/or coach’s confidence in the competence of his/her medical support staff. In recent years, a subtype of hamstring muscle injury—involving disruption of the intramuscular tendon (IMT) (an intramuscular continuation of the proximal and distal free tendons) [23, 24]—has been posited to associate with the high recurrence rates of hamstring muscle injuries [23]. Despite IMT disruption being present in up to 41% of all hamstring muscle injuries [25], there is currently a lack of data to guide clinicians in managing these injuries [26].

Differences in therapeutic rehabilitation strategies and reported outcomes in the published literature add to the confusion [17, 25, 27–29]. Many of the reported cases in the published literature have come from high-performance sport, where these injuries pose a particular challenge. Accurate prognoses are imperative to facilitate team selection and tactical planning in high-performance sport. Considering the importance of mitigating the risk of injury recurrence and optimising athletes’ recovery, it is necessary to understand the relevance of the injured anatomical structure [30]. The purpose of this review is to examine whether hamstring muscle injuries involving injury to the IMT warrant being classified as a distinct clinical entity with expected different outcomes to other hamstring muscle injuries.

## Methods

While this is a narrative review, a systematic search was performed on the PubMed database (from inception to March 2022). The keywords used for the search were derived from the research question (Table 1). The titles and abstracts of all articles identified by the search were screened independently by two authors (FK and DC). Full text screening was undertaken in cases whereby it was difficult to determine whether the study should be included from the title and abstract alone. Studies were included if they reported on acute hamstring muscle injuries involving the IMT incurred by athletes (Additional file 1). Two authors (FK and DC) extracted data relating to the key results, study population, rehabilitation approach and length of athlete tracking post-injury.

**Table 1:** Keywords used in the literature search

Concept	Population	Hamstring	Pathology/injury	Location
Keywords	Athlet* Sport\Exercise	Biceps femoris	Tear	Intramuscular tendon
		Semitendinosus	Strain	Central tendon
		Semimembranosus	Injur*	Aponeurosis
		Hamstring*	Re-injur*	Intratendinous
		Posterior thigh		

*Truncation

## Result

### Intramuscular Tendon of the Hamstring Muscles

The tendons of the hamstring muscles are composed of distinct components—the proximal and distal free tendons, so-named because they are devoid of inserting muscle fascicles, and the IMT to which muscle fibres are attached. The IMT (also called the ‘central tendon’, ‘aponeurosis’, or ‘intramuscular connective tissue’) is a continuum of the free tendon, which extends within the muscle belly [24], akin to the central rachis of a feather, with radiating myofibrils [23].

This arrangement functions as a central supporting scaffold to which the muscle fibres attach. This connection point, or musculotendinous junction, is the point at which most hamstring muscle injuries occur [32, 33]. However, it has increasingly been recognised that injury can extend into the IMT itself, with recent studies demonstrating disruption of the IMT in 19–41% of hamstring muscle injuries (Table 2) [25, 27–29].

**Table 2:** Comparison of outcomes following intramuscular tendon injury

	Comin et al. [28]	Eggleston et al. [17]	Lempainen et al. [43]	McAuley et al. [63]	Murphy and Rennie [26]	Pollock et al. [29]	Pollock et al. [55]	Shamji et al. [62]	Taberner and Cohen [61]	van der Made et al. [25]	van der Made et al. [27]	Vermeulen et al. [60]
IMT injuries	12	16	8	6	1	15	13	13	1	29	64	41
Cohort	AFL and RL	AFL	Mixed—mainly soccer	Single EPL team	EPL footballer	Elite T + F	Elite T + F	Single EPL team	EPL footballer	Mixed—mainly soccer	Mixed—mainly soccer	Mixed—mainly soccer
Conservative management	9	16	0	Unclear	0	0	13	Unclear	1	29	64	41
Surgical management	3	0	8	Unclear	1	15	0	Unclear	0	0	0	0
Recurrence	n/a	31%	0%	n/a	Unclear	60%	0%	16%	0%	17%	20%	20%
2c	n/a	n/a	n/a	n/a		63%	0%		n/a	0	n/a	n/a
3c	n/a	40%	n/a	n/a		57%	0%		n/a	21%	n/a	n/a
Blinded to MRI findings	No	No	No	No	No	No	No	No	No	Yes	Yes	Yes
Location												
BF	100%	n/a	62%	n/a	100%	n/a	85%	100%	n/a	59%	75%	56%
SM	0%	n/a	25%	n/a	0%	n/a	15%	n/a	n/a	17%	13%	20%
ST	0%	n/a	0%	n/a	0%	n/a		n/a	n/a	24%	13%	0%
BF/ST	0%	n/a	13%	n/a	0%	n/a		n/a	n/a	0%	0%	24%
TTRTP/TTRFT												
Conservative	72 (IQR 42–109)	21–105	n/a	n/a	n/a	54 days	37.5	37 (8–103)	120 days	28 ± 10 days	n/a	31 (IQR 22–42)
Surgical	91 days (IQR 84–91)	n/a	3.5–4.5 months	n/a	10 weeks	n/a	n/a	n/a	n/a	n/a	n/a	n/a
2c	n/a	n/a	n/a	n/a	n/a	27 ± 6.8 (Range 18–35)	35 (IQR 9.5, Range 25–43)	n/a	n/a	24 days	n/a	n/a
3c	n/a	n/a	n/a	n/a	n/a	84 ± 49.4 (Range 40–128)	51.5 (IQR 23.8, Range 28–70)	n/a	n/a	28 days	n/a	n/a
Rehabilitation	n/a	Criteria based	Running based, no strength	Criteria based	Criteria based—strength and field	No formal criteria	BAMIC' approach	Strength and function based	Criteria based	Criteria based	Criteria based	Criteria based
Follow-up	Unclear	Unclear	8–24 months	12 months	Unclear	3 months	3 months	3 months	13 months	12 months	12 months	12 months

*IMT* Intramuscular tendon, *EPL* English Premier League, *AFL* Australian Football League, *RL* Rugby league, *T* + *F* Track and field, *BF* biceps femoris, *SM* semimembranosus, *ST* semitendinosus, *TTRTP* Time to return to play, *TTRFT* Time to return to full training, *n/a* Unclear or not specifically included, *IQT* interquartile range

Although the collagenous structure of the IMT has similarities to that of the free tendons [31], there are critical differences. In some respects, the IMT could be considered a transitionary structure, as the arrangement of the collagen-proteoglycan matrix is more wavy and disorganised than in free tendons [24]. In addition, the cross-sectional area of the IMT may be smaller and stiffer (strains of 2% in IMT versus 6% in the energy storing free tendons, allowed by interfascicular gliding) [34, 35].

The IMT also differs from free tendon in its relationship to pathology. Free tendon pathology is associated with overuse and degeneration, with acute tears being less frequent. Perhaps due to their greater vascularity, such a pattern is not observed at the IMT where injuries tend to be acute strains. These acute injuries are likely to be the result of the stress applied at the adjacent musculotendinous junction, which is much larger at the IMT than the free tendon [31]. These differences suggest the need for clear delineation between injured structures when considering optimal loading, tissue healing and rehabilitation [36].

The anatomical differences between the tissues could impact upon its elasticity and would indicate that the IMT does not store and release energy in the same way that free tendons do [31]. Should this be the case for the IMT of the hamstring muscles, then this would have implications for management of these injuries, particularly concerning the prioritisation of plyometric exercises and sprinting in rehabilitation [37].

### Mechanisms of Intramuscular Tendon Injury

The comprehensive injury-causation model developed by Bahr and Krosshaug [38] outlines the interaction of internal and external risk factors in the occurrence of sport-related injuries; it is based on the epidemiologic model of injury developed by Meeuwisse [39] and the biomechanical model of injury proposed by McIntosh [40]. The comprehensive injury-causation model [38] furthers these two models by highlighting the importance of injury mechanisms. Qualitatively detailing the activities during which injuries occur (e.g. landing from a jump), and the biomechanics (e.g. kinematics/movement profiles) of the injury event can provide clinicians with useful information to help guide them in their assessment of potentially injured tissues.

However, such descriptions of the mechanisms of hamstring muscles injuries are lacking in the published literature. Askling et al. [41, 42] have proposed that two distinct types of hamstring muscle injuries are most prevalent in sports, which they termed—‘sprint’ and ‘stretch’ types. They reported that injuries to the proximal free tendon of semimembranosus were more likely to be sustained during the ‘stretch’ type of hamstring muscle injury [41, 42]. Interestingly, these authors do not refer specifically to the IMT.

In a published case series documenting surgical/operative treatment of IMT injuries, Lempainen et al. [43] reported that the mechanism of injury in all included cases was “similar to that of the typical hamstring injuries: a rapid change in direction during fast sprinting or overstretching of the hamstring while falling”. More recently, Eggleston et al. (2020) reported that all hamstring muscle injuries which involved the IMT included in their retrospective study were sustained during sprinting [17].

A study describing IMT injuries in Australian Rules football players observed that all injuries occurred during sprinting, with none sustained during stretching [17]. However, such binary, linear descriptions only consider the injury as occurring in one plane of movement and may lack the detail to truly assist in prognosis and diagnosis of these challenging injuries. For instance, it is possible that a combination of a ‘sprint’ and ‘stretch’ type injury could occur in tandem, or that specific activities or positions may disrupt the IMT. Our group have previously described positions of trunk rotation and trunk flexion at the likely time of hamstring strain injuries in professional rugby union players [44]. Where possible, it would be worthwhile in further analyses of IMT injuries for authors to provide greater detail in descriptions as to the biomechanical position of athletes at the point of injury, in order to determine whether there is a distinct, identifiable pattern of injury.

### Tendon Healing

Free tendons are stronger and stiffer than muscle, and function differently [45]. Muscle and tendon undergo different repair and regeneration processes following injury. This contributes to the prolonged time to return to play (RTP) following proximal free tendon injury when compared to time to RTP following musculotendinous junction injury [12]. Muscle healing is characterised by satellite cell proliferation which provides a scaffold for myofiber regeneration [33]. This can occur within the first week following injury, allowing for an accelerated return of function, particularly when the compromise of the contractile capacity of the tissue is minimal [37]. In contrast, tendon repair is slow and initially produces a mechanically inferior scar to provide continuity across the injured site in the proliferation phase [46]. This scar is primarily comprised of type III collagen arranged randomly within the extracellular matrix. Tendon repair and regeneration is a complex process; it requires gliding and loading to prevent adhesions and to stimulate a healthy scar. The remodelling phase begins 6–8 weeks after injury, facilitated by mechanotransduction in structured rehabilitation, where repeated loading stimulates deposition and remodelling of the collagenous matrix, providing mechanical strength to the tissue [46].

Though these differences in regeneration explain the variance in recovery between proximal free tendon injury and muscle injury, it is not yet clear whether these processes have implications for IMT management. Certainly, it makes theoretical sense that the histological commonalities between IMT and free tendon may underpin the greater recurrence rates and prolonged recovery often reported following IMT injury than with myofascial or musculotendinous junction injury [17, 28, 29]. However, the differences in organisation of the collagen structure of the IMT, allied to its adjacent muscular scaffold could explain why IMT injuries are less severe than free tendon injuries [30, 47].

The role of the well-organised tendon is to transmit large tensile forces, meaning adequate repair would be required to restore capacity for dynamic tasks in sport, such as sprinting and kicking. However, given the differences in morphology between the free tendon and the IMT [23] and overlap in outcomes between muscle injuries and those involving the IMT [25, 27], it is not clear if this supposition is accurate.

There is surprisingly little direction for specificity in management approaches for these injuries, with only Macdonald et al. [37] describing a bespoke rehabilitation paradigm which gives consideration to tendon-specific loading. However, there remains a great degree of unknown as to what optimal loading means for these injuries [36], and whether the principles of free tendon healing or tendinopathy rehabilitation have relevance to IMT injuries remains uncertain.

### Magnetic Resonance Imaging (MRI)

Across sports, MRI is frequently used to investigate acute and recurrent HSIs [48, 49]. It is an accurate and reliable method of defining the injured structure and degree of tissue damage [50]. However, a systematic review by Reurink et al. [49] reported a lack of strong evidence for any MRI finding with predictive association for time to RTP following HSI. This was due to a sizeable risk of bias in 11 of the 12 included studies. More recently, a retrospective analysis of 59 professional athletes showed that baseline MRI alone was not useful in predicting re-injury [51]. However, the baseline MRI findings were used to tailor the rehabilitation processes and later used for comparison with a further MRI before RTP clearance.

However, despite the inconsistent evidence that the MRI findings influence recovery, in practice, imaging continues to play a considerable role in the management of hamstring muscle injuries.

At that time point, only Comin et al. [28] had discussed the relevance of IMT injury in prognostication. On MRI, a disrupted IMT may have a wavy, retracted appearance, compared with the taut, cord-like appearance of an uninjured structure. It may also be seen to retract following injury, creating a gap. Driven by the need to advance the predictive ability of MRI, novel classification systems have been proposed in recent years [52]. The British Athletics Muscle Injury Classification (BAMIC) has been designed specifically for hamstring muscle injuries, although it has been proposed that this system can be applied to other injury sites [53]. This system categorises injury by degree of damage, but also using an alphabetical scale where IMT injuries (classified ‘c’) are the most severe. This has been shown to be a reproducible diagnostic framework, and initial work suggested that ‘c’ type injuries were associated with a greater risk of recurrence and time to return to full training [29]. However, there remains wide overlap and variance between the categories of injuries so far, and it is not yet clear whether such MRI grading systems can accurately predict time to RTP or the likelihood of injury recurrence.

Recent promising evidence may help with the evolution of MRI interpretation in guiding the late stage of rehabilitation. Isern-Kebschull et al. [54] carried out MRI imaging within 7 days of professional athletes returning to play following HSI and found that transversal and/or mixed connective tissue gap, loss of tendon tension, intermuscular oedema, callus gap and interstitial feather oedema pattern, was associated with a high risk for reinjury when two of these findings, were present (OR 29.58; 95% CI 3.86–226.64; *p* = 0.001). In elite sport where access to imaging is readily available, these data may greatly influence future decision making. The influence of rehabilitation strategy on promoting these determinants of healing is unknown, but conceivably, this could explain the success of rehabilitation approaches that take an approach to exercise designed to promote early protection of the IMT [55].

### Clinical Assessment of Intramuscular Tendon Injury

It has been suggested that the use of MRI is imperative in the assessment of hamstring strain injuries, as it is otherwise impossible to distinguish between injuries which do and do not involve the IMT [37]. In a cross-comparison of clinical and MRI findings, Crema et al. [56] reported that participants with IMT disruption had reduced flexibility (*p* = 0.006) and strength compared to those participants without IMT disruption [56]. However, the utility of these results in day-to-day clinical practice is compromised by the high degree of overlap and variance between injuries, meaning that it would be impossible to determine which injuries do and do not involve the IMT.

Also, it is important to consider that the study by Crema et al. was cross-sectional in design; meaning that it cannot be directly inferred that decrements in flexibility and strength were solely the consequence of IMT injury. This adds further to the diagnostic challenge that exists around these injuries.

Given these injuries likely benefit from bespoke management, it would be useful to able to determine the likelihood of IMT injury using clinical assessment tests to determine prognosis without the use of imaging, or to select athletes for whom imaging is indicated. In fact, as these injuries may initially present better than may be suspected for such a ‘serious thigh injury’, clinicians may be more likely to overlook the presence of IMT injury. This diagnostic blind spot may be implicated in the high recurrence rates, particularly if athletes are progressed at an inappropriate rate through their rehabilitation process.

### Non-operative Outcomes

In a recent review, Brukner [57] advocated that a high suspicion of IMT injury should accompany any hamstring muscle injury recurrence. In this review, the author presents the viewpoint that injuries involving significant IMT disruption require prolonged rehabilitation and may be more prone to recurrence. However, the evidence to support this viewpoint is mixed, with recent indications suggesting that there is minimal meaningful difference in re-injury rate or time to RTP between these (IMT) injuries and other ‘typical’ hamstring muscle injuries (Table 3) [25, 27, 52].

**Table 3:** Comparison of time to return to play and recurrence rates between hamstring strain injuries that involve disruption of the intramuscular tendon and those that do not

	Pollock et al. [29]	van der Made et al. [25]	van der Made et al. [27]
*Recurrence rate*			
IMT injury	60%	17%	20%
Other HSI	8%	15%	20%
Association between IMT and increased risk	*p* = 0.001	n/a	*p* = 0.898
*Time to RTP*			n/a
IMT injury	54 days	28 days	n/a
Other HSI	20	22 days	n/a
Association between IMT and increased time to RTP	*p* = 0.08	*p* = 0.025 (following full thickness)	n/a

*IMT* Intramuscular tendon, *HSI* hamstring strain injury, *RTP* return to play

The earliest study to compare outcomes between IMT disruption and other hamstring muscle injuries not involving the tendon was undertaken by Comin et al. [28]. The authors retrospectively analysed injury databases from 6 Australian Rules football teams and one professional rugby league team. Of the 62 hamstring muscle injuries included in the study, 12 involved the IMT. All of these injuries were to the biceps femoris muscle, with none involving any other hamstring muscle. The authors reported that IMT injuries were significantly more likely to recur than other injuries. Additionally, the mean recovery time following IMT disruption was more than 3 times (72 days; interquartile range [IQR]: 42–109) that of other biceps femoris muscle injuries (21 days; IQR 9–28), as well as 3 times that of the median combined recovery time of injuries to all hamstring muscles (21 days; IQR: 14–42).

Similarly, Pollock et al. [29] described 14 cases of IMT in elite track and field athletes between 2010 and 2014. Injuries that involved the IMT had substantially greater risk of recurrence at 3 months follow-up (57% recurrence following 2c injury and 63% following 3c injury). These were also associated with a significantly longer time to return to full training (the most appropriate clearance metric in this cohort), with athletes absent for a mean period of 54 days (2c: 27 ± 6.8 days, 3c: 84 ± 49.4 days). Six cases (27%) incurred exacerbations, or reinjuries during rehabilitation, compared to just 4% of other hamstring muscle injuries. No other study describes exacerbation rate during rehabilitation, and it is unclear if this is specific to this particular group, rehabilitation philosophy, or sport. The authors proposed that the high recurrence rate is due to the physical demands of sprinting, particularly with the high speeds involved in elite track and field—this is an appealing conclusion given that it has been recently demonstrated that no rehabilitation exercise can match the high activation demands of sprinting [58]. However, the authors did not state what the mechanisms of injuries were, so it is unclear if these reinjuries and exacerbations actually occurred during sprinting.

Responding to these critically high recurrence rates, this group re-evaluated their rehabilitation approach and published bespoke principles specific to the BAMIC framework. They subsequently reported no recurrences of 13 IMT injuries between 2015 and 2019, and just 3% for all hamstring muscle injuries. Time to return to full training was significantly longer following IMT injury than other injury locations. However, the athletes’ recovery time was broadly similar to these authors' previous work (2c injury: mean = 35 days, 3c injury: 51.5), suggesting that it is the difference in rehabilitation that defines the outcomes, rather than just longer time allowed for healing. However, it is critical note that these results—particularly regarding time to return to play—may be to specific to the sport of track and field and may not be generalisable to other sports with different physical demands and mechanisms of injury [44, 59].

Wangensteen et al. [52], as part of a larger prospective analysis of hamstring muscle injuries, demonstrated only a small difference in time to RTP following IMT injury compared with other types of HSI. However, given the wide overlap and variance between the time to RTP amongst injury grades, the authors concluded that this small difference in time to RTP would not be useful in guiding prognosis. The BAMIC categorisation of an injury only accounted for 7.6% to 11.9% of total variance in time to RTP. Although the parties in this study were not blinded to the grading and classification, the rehabilitation model was standardised, rather than tempered or modified due to the presence of IMT disruption.

Two further studies prospectively investigated outcomes following hamstring muscle injuries in athletes, primarily professional footballers across two centres in the Netherlands and Qatar [25, 27]. These were large prospective studies, which used standardised clinical criteria for rehabilitation progression (distinct to each centre). These are also the only studies in which clinicians and participants were blinded to MRI findings, and thus may give the most accurate reflection of recovery time and injury recurrence following IMT injury. Despite reported recurrence rates of 17–20% [25, 27], there was no difference between IMT injuries and typical hamstring muscle injuries [27]. Participants with waviness on MRI and full thickness disruption of the IMT had a mean increase in time to RTP of 9 days. While in sport, 9 days can be costly to an athlete or team, the between group variance and overlap was such that the authors cautioned against interpreting this as inferring predictive capacity. However, while Pollock et al. [29] provided follow-up at three months, these studies included a 12-months follow-up. Retrospective post-hoc analysis did demonstrate an increased re-injury rate following IMT compared to other hamstring muscle injuries at 3 months, but the study was inadequately powered to infer significance. This does, however, suggest that the risk of failure could be greatest in the early stage, perhaps due to the vulnerability of the remodelling tissue.

Within a similar cohort of Dutch and Qatari athletes, Vermeulen et al. [60] investigated the associations between IMT disruption at initial MRI, level of resolution at the time of RTP clearance and re-injury risk. Having followed a standardised rehabilitation process, and completed at least 5 days of unrestricted training before RTP clearance, 20% of included participants incurred a re-injury at 12 months follow-up. However, Vermeulen et al. [60] have shown that 56% of athletes had a measure of discontinuity (1 complete, 22 partial) on RTP, with a similar propensity for recurrence as those who did not. None of the seven participants with complete IMT discontinuity incurred a re-injury during the study follow-up time period.

In addition to the difference in follow-up (3 months versus 12 months), a differentiating characteristic of the cohorts [25, 27, 60] is that the clinicians and athletes were blinded to MRI findings. This eliminates any potential bias regarding IMT injuries. In all other cases, findings may be biased by the availability of MRI results. However, in clinical practice, particularly in elite sport, it is inconceivable that the involved parties could be blinded. This cultural phenomenon is in spite of the equivocal utility of current MRI grading systems to assist with RTP prediction [52], and the questionable relevance of IMT healing on MRI at RTP [60]. This does raise the question, however, of whether clinicians’ or athletes’ expectations are influenced by their beliefs that IMT injuries are a more significant type of HSI.

A single case report has described the rehabilitation of an English Premier League footballer [61]. The authors suggest the use of strength and rate of force development monitoring to support the return to dynamic tasks associated with match play. However, the authors did not specify which muscle was injured, which may limit the transferability of some aspects of this approach. The player in this case was cleared to RTP 120 days following injury and had no recurrence during a 13-month follow-up. It is possible that the thorough use of outcome measures described by the authors contributed to the prolonged time to RTP.

Two recent single English Premier League soccer club retrospective observational studies describe the impact of IMT involvement on time to RTP and reinjury. Shamji et al. [62] reported significantly longer time to return to full training following IMT injury compared with other hamstring muscle injury types (36 days compared to 24 days), although there were just three BAMIC grade 3c injuries included. McAuley et al. [63] reported no differences in time to RTP or injury recurrence between injuries with and without tendon involvement of the same grade. However, injuries that were exacerbated during rehabilitation were excluded from final analysis of time to RTP. In addition, this study analysed just 6 IMT injuries, none of which were categorised as BAMIC grade 3c. Both of these studies describe recovery times that are more comparable to those reported by van der Made et al. [27] than those described by Pollock et al. [29], possibly suggesting differences in outcome are mediated by the injured athlete’s sport as well as severity.

It may be the case that in sports such as football, athletes can continue to compete while not sprinting maximally [64], and that the top speeds and forces of these athletes are likely to be less than the elite track and field athletes in the cohort included in the study by Pollock et al. [29] However, as few as 60% of IMT injuries may actually occur during sprinting [52]. Also, a greater proportion of hamstring muscle injuries involved the IMT in studies including athletes from sports other than track and field (23% compared to 39–41%) [25, 27, 29]. Thus, it is unclear if the variation in injury outcomes can be explained simply by differences in running speed across sport.

A recently published series details the outcomes following IMT injuries in Australian Rules football players at a single club over five year period [17]. Though the clinicians and athletes were not blinded to the results, the BAMIC classification was not in use at the club at the time. Thus the clinicians did not deliberately modify rehabilitation based on the presence of IMT disruption but instead responded to clinical progression. Injury recurrence rates of 31% were reported following IMT, compared to 12% following typical hamstring muscle injury. These authors advocate a further subclassification of IMT injuries depending on the location, degree of injury and number of muscles involved. Although Pollock et al. [29] did not demonstrate poorer prognosis following proximal IMT injury, this is worthy of further investigation as deeper classification may explain the observed variance in results across published studies.

### Rehabilitation

Appropriately staged rehabilitation is pivotal to positive outcomes following any sport-related injury. It appears that this may be particularly the case following IMT injury [37, 55]. While similar time to RTP and recurrence rates to other locations in the musculotendinous unit have been demonstrated [25, 27, 52, 55], the variation and bias in rehabilitation processes implemented by authors may explain the variation in results. For example, as the clinicians were blinded to MRI results during the administration of the rehabilitation protocol in the cohorts described by van der Made et al. [25] van der Made et al. [27] and Vermeulen et al. [60], they were unable to apply distinct management principles that considered specific IMT loading.

A logical and well-reasoned paradigm proposing delayed lengthening and eccentric activation in response to the specific structural injury has been suggested by Macdonald et al. [37] where progression and exercise selection that is ‘intramuscular tendon-oriented’ relates to the principles of tendon healing and function.

Practically applying this approach, Pollock et al. [55] demonstrated a particularly low reinjury rate in their elite track and field cohort (2.9%, with no reinjuries involving the ‘c’ class injuries). This compares more favourably with any other exercise-based therapeutic approach suggested for hamstring injury rehabilitation, given that other similarly low recurrence rates in the lengthening protocol described by Askling et al. [12], and the criteria-based approach described by Mendiguchia et al. [11] had longer time to return to full training, or injuries of lesser severity, respectively. As the time to return to full training described by Pollock et al. [55] is similar to the authors’ previous published results, it is plausible that the improved outcomes are a result of the changes in the loading protocol. However, it is unclear whether there are decrements in performance associated with a more cautious approach to rehabilitation. In addition, it would be necessary to see this approach applied to larger groups and across multiple sports and genders.

While it has been shown that it is possible to progress through rehabilitation while tolerating some pain during exercise and running without compromising outcomes [10], De Vos et al. [65] demonstrated that athletes returning to sport with ongoing strength and flexibility deficits and pain were significantly more likely to incur a reinjury. In this instance it was not possible to ascertain the interaction between rehabilitation design, injury location, and injury recurrence. It may be that in sports such as track and field where work demands on the hamstring muscles increase in a nonlinear manner as speed increases [66], there is less room for error following IMT injury. Also, despite not demonstrating a difference in recurrence rates between groups, reinjury rates of 17–20% were reported following IMT by van der Made et al. [25] van der Made et al. [27]. It is possible that these could be reduced further with the integration of a more specific framework which considers injury location [55].

Additionally, it has been reported that a smaller proximal biceps femoris IMT may be a risk factor for HSI [67]. Given hypertrophy training has been shown to impact IMT size in other muscle groups [68], Bourne et al. [69] posited that this may explain some of the protective effect of resistance training in HSIs. However, this hypothesis requires further investigation generally, as well as specifically for IMT injuries.

Furthermore, as no specific description of the mechanism of IMT injury has been described, it is unclear whether there are specific modes of assessment or rehabilitation that could be integrated to better prepare athletes to RTP. Biomechanical positions or tasks associated with injury could form part of a rehabilitation pathway, in order to better prepare athletes for the demands of the injurious actions associated with their sport, as well as giving the athlete confidence at the point of RTP.

Several of the series describing these injuries do not describe progressive, criteria-based rehabilitation [28, 29], despite such approaches now forming the gold-standard in rehabilitation, particularly in elite sport [11, 70]. In addition, the use of strengthening exercises has not been described following surgical repair [43]. As strengthening exercises have been shown to induce specific adaptations following injury [69], these seem crucial for inclusion in any rehabilitation programme. Additionally, a lack of evidence-informed guidelines to assist clinicians has the potential to compromise post-surgical outcomes, particularly as the rarity of IMT repair surgery means many clinicians will have little experience rehabilitating these injuries.

In addition, no prospective data are available which tracks the recovery of aspects of neuromuscular function following IMT injury. While there is evidence that eccentric strength, electrical activity, fascicle length, and sprint performance are impacted following HSI [19, 69, 71, 72], this has not been demonstrated to be specific to injury location. Considering that a high proportion of hamstring muscle injuries involve injury to the IMT, it would be worthwhile to note whether some of these neuromuscular impairments are specific to injury location, or whether their recovery occurs at differing timepoints following injury.

### Surgery

Though operative repair is a recognised management for severe IMT injuries, there is a paucity of information to guide post-operative rehabilitation practices [28]. Operative repair is the strategy of choice for avulsions or complete free tendon ruptures [73]. For injuries to the IMT, however, the current trend does appear to tend towards conservative management, given the recent positive findings published by Pollock et al. [55]. Several other authors have advocated non-operative rehabilitation akin to a typical HSI [25, 27, 29, 55]. However, Lempainen et al. [43] detailed a case series of 9 athletes, including professional soccer players, with reported excellent results following surgical repair and RTP times ranging from 3.5 to 4.5 months. Comin et al. [28] reported upon 3 cases of Australian professional athletes who underwent surgical repair, with an average time to RTP of 91 days (interquartile range 84–91).

Surgery may be the best option following IMT injury in chronic cases where the athlete has incurred multiple recurrent injuries that are impacting upon his/her performance, or where there is a clear gap between the IMT ends [43]. However, there is no clear consensus on whether gapping is an indication for surgery, or whether there is a gap threshold beyond which surgery is indicated. Interestingly, no grade 4 injuries were evaluated by Pollock et al. [55].

In the case series by Lempainen et al. [43] the surgical procedure was described in detail. The aim of the surgery is to restore the tension of the IMT, while taking care not to repair the tissue too tightly. The patient is positioned prone with the knees in slight flexion to decrease tension within the hamstring muscles. A vertical incision and fasciotomy is made over the injured area to allow the ruptured central tendon to be identified. The authors warn that the proximity of the sciatic nerve to the likely injured site should be noted, as this may be injured during the procedure. It is possible that neural scarring or tethering could be a potential cause of lingering symptoms or dysfunction, and it should be considered a differential diagnosis in stubborn cases.

With advances in medical imaging, and wider subcategorization within the classification scales, it may become clearer which injuries are more likely to be refractory to conservative management. It has been suggested that severe proximal IMT injuries or those involving more than one muscle may be at risk of higher recurrence rates [17], and this may be worthy of investigation in future analyses to determine whether these would benefit from surgery as a primary approach.

There is little evidence to guide the specifics of post-operative rehabilitation. Comin et al. [28] involved cases across multiple centres, with no details provided about the post-operative rehabilitation implemented. Lempainen et al. [43] advocate a protective phase. During this phase, the athlete is advised to avoid stretching and prolonged sitting, and to weight-bear as tolerated on crutches. The use of a brace is not advocated in this phase. The athlete then advances to a rehabilitation phase involving aqua-training, anti-gravity treadmill running and a four-step field-based running protocol. While this model may have since evolved, no pathway for the introduction of strengthening exercises has been described, and this should be a central component of any rehabilitation pathway to return the athlete to full function and minimise impairments upon RTP [69]. As a result, given that it is clear that progressive, criteria-based rehabilitation models are necessary to optimise recovery, clinicians remain uninformed as to how to periodise hamstring strengthening and rehabilitation following IMT surgical repair.

Though prolonged healing is required for the development of a functionally mature scar following tendon injury, the restoration of anatomy during surgical procedure could aid early progression and loading At other sites, accelerated RTP following the surgical repair of a tendon avulsion has been demonstrated [74], where the athlete followed a criteria-based rehabilitation programme. However, the median time to RTP following surgery reported by Comin et al. [28] was 19 days longer than non-operative management. At present, there is insufficient evidence to ascertain what the optimum approach to rehabilitation is, and whether RTP timeframes could be reduced following this procedure or whether surgery is reserved for more severe cases. Clarity would be particularly useful in elite sport, where the requirements of the team and the player to optimise availability may influence clinical management. More extensive case series are required to further expand understandings of these injuries, as at present, the low numbers of these cases reported in the literature prevent consensus on whether expedited outcomes can be achieved in certain cases. In addition, without randomised controlled trials to compare conservative and surgical management of high grade injuries, it is probable that these injuries will continue to be influenced by the preferences, attitudes and cultures of the athletes’ environments rather than empirical evidence.

## Conclusion

There are anatomical differences between the IMT and free tendon, and these may impact function and recovery following injury. Tissue damage to the IMT may be observed on MRI following acute hamstring muscle injury, using novel grading systems. Though prolonged time to return to play and higher injury recurrence rates have been reported for injuries to the IMT, the evidence is conflicting. Variance in the injury site and rehabilitation strategies described may be implicated in the heterogeneity of outcomes.

There is some indication that early protection may assist in the reduction in recurrences. However, further research aimed at investigating the effectiveness of such criteria-based rehabilitation interventions across multiple sports would add greatly to our understanding of this subtype of hamstring muscle injury.

### Supplementary Information

**Additional file1.** Search Strategy and eligibility criteria.

## Data Availability

Not applicable.

## Abbreviations

HSI: Hamstring strain injury
IMT: Intramuscular tendon
TTRTP: Time to return to play
RTP: Return to play
MRI: Magnetic resonance imaging
