# Supplementary material for: Intramuscular Tendon Injuries of the Hamstring Muscles: A More Severe Variant? A Narrative Review
Source: Sports Med Open. 2023 Aug 14;9:75. doi: 10.1186/s40798-023-00621-4 (PMC10425319; doi:10.1186/s40798-023-00621-4)
Supplement: Supplementary file 1 — Additional file1. Search Strategy and eligibility criteria. [file 40798_2023_621_MOESM1_ESM.docx]

**Search Strategy and eligibility criteria:**

A systematic search was performed on the PubMed database (from inception to March 2022). The keywords used for the search were derived from the research question (do intramuscular tendon injuries warrant being classified as a distinct clinical entity with expected different outcomes to other hamstring muscle injuries?). These are listed in Table 1.  

The titles and abstracts of all articles identified by the search were screened independently by two authors (FK and DC). The reference lists of each study considered were screened for potential studies of interest that had not previously been identified.  Full text screening was undertaken in cases whereby it was difficult to determine whether the study should be included from the title and abstract alone. Studies were included if they reported on acute hamstring muscle injuries involving the IMT incurred by athletes.

All the original studies that investigated rehabilitation outcomes following IMT injury were evaluated for eligibility. Studies describing hamstring injuries involving the proximal or distal free tendons were not considered. Studies were included if they were in English, and conclusions described the outcomes following hamstring strain injuries. Relevant editorials, literature reviews, systematic reviews, meta-analyses and chapters from textbooks were analysed and summarised to provide context and background.

Two authors (FK and DC) extracted data relating to the key results (time to return to full training and recurrence rate), study population, rehabilitation approach and length of athlete tracking post-injury. Potential sources of bias, including participant and clinician blinding, where present, were also noted. The authors also summarised the discussion and conclusions of each study. This process was performed in duplicate by these authors using a Microsoft Excel (Microsoft, USA) spreadsheet.
